# Berufsvorbereitung und Präferenzen für Tätigkeitsfelder von Studierenden der Hebammenwissenschaft in Deutschland

**DOI:** 10.1055/a-2625-2758

**Published:** 2025-07-10

**Authors:** Caroline Johanna Agricola, Ilona Efimov, Matthias Augustin, Volker Harth, Albert Nienhaus, Stefanie Mache, Birgit-Christiane Zyriax

**Affiliations:** 1 37734 Hebammenwissenschaft – Versorgungsforschung und Prävention, Institut für Versorgungsforschung in der Dermatologie und bei Pflegeberufen (IVDP), Universitätsklinikum Hamburg-Eppendorf (UKE), Hamburg, Germany; 2 37734 Zentralinstitut für Arbeitsmedizin und Maritime Medizin (ZfAM), Universitätsklinikum Hamburg-Eppendorf (UKE), Hamburg, Germany; 3 37734 Institut für Versorgungsforschung in der Dermatologie und bei Pflegeberufen (IVDP), Universitätsklinikum Hamburg-Eppendorf (UKE), Hamburg, Germany

**Keywords:** Arbeitsplatzwahl, Berufsvorbereitung, Hebammenwissenschaft, Tätigkeitsfelder, First job choice, Preparedness for professional practice, Midwifery, Fields of practice

## Abstract

**Hintergrund:**

Studierende der Hebammenwissenschaft werden durch das Studium für die Versorgung von Frauen und ihren Familien befähigt. Mit der Akademisierung im Jahr 2019 wurde die theoretische Ausbildung von Berufsschulen an Hochschulen überführt, um den steigenden Anforderungen an den Beruf gerecht zu werden. Ziel dieser Studie ist es, die subjektive Vorbereitung auf die Berufsausübung und die interprofessionelle Zusammenarbeit sowie die Präferenzen für Tätigkeitsfelder aus Sicht von Studierenden der Hebammenwissenschaft zu untersuchen.

**Methodik:**

Die Querschnittsstudie „Healthy MidStudents“ zur Untersuchung der Gesundheits- und Arbeitssituation von Studierenden der Hebammenwissenschaft ( *n* =342, Rücklaufquote 61,3%) wurde in Norddeutschland an neun Studienstandorten zwischen dem 17.10.2022 und dem 31.01.2023 durchgeführt. Ausgewertet wurden die Daten deskriptiv und inferenzstatistisch.

**Ergebnisse:**

Der Großteil der primärqualifizierenden Studierenden ( *n* =249) fühlten sich sowohl für die Berufsausübung (47,0%) als auch für die interprofessionelle Zusammenarbeit (47,4%) im Durchschnitt mittelmäßig vorbereitet. Die Tätigkeit in der außerklinischen Geburtshilfe (24,3%), gefolgt von der klinischen Geburtshilfe (21,6%) und der außerklinischen Wochenbettbetreuung (14,6%) wurden von Studierenden ( *n* =342) am häufigsten als präferierte Tätigkeitsfelder gewichtet.

**Schlussfolgerungen:**

Die Ergebnisse implizieren Maßnahmen, zum Beispiel Mentoring Programme, um die subjektive Vorbereitung auf die Berufsausübung und die interprofessionelle Zusammenarbeit zu stärken. Die Studienergebnisse zeigen, dass ein breitgestreutes Interesse für verschiedene Tätigkeitsfelder, insbesondere für außerklinische Tätigkeitsfelder, besteht.

## Abstract (en)

**Background:**

Student midwives are trained to provide care for women and their families. With academisation in 2019, theoretical education was transferred from vocational schools to institutions of higher education in order to meet the increasing demands of the profession. The aim of this study is to examine the preparedness for professional practice and interprofessional collaboration as well as preferences in fields of practice of student midwives.

**Method:**

The cross-sectional study “Healthy MidStudents” examining the health and working conditions of student midwives ( *n* =342, response rate 61.3%) was conducted in Northern Germany at nine institutions of higher education between 17 October 2022 and 31 January 2023. The data was analysed using descriptive and inferential statistics.

**Results:**

The majority of primary-qualifying students ( *n* =249) rated that they were on average moderately prepared for both the professional practice (47.0%) and interprofessional collaboration (47.4%). Students ( *n* =342) most preferred working in out-of-hospital birth care (24.3%), followed by clinical birth care (21.6%) and out-of-hospital postnatal care (14.6%).

**Conclusions:**

The findings implicate measures, e.g. mentoring programmes, to promote preparedness for professional practice and interprofessional collaboration. The study results indicate a broad interest in various fields of practice, particularly in out-of-hospital fields.

## Hintergrund

Seit dem Jahr 1985 erfolgte die Ausbildung zur Hebamme in Deutschland im Rahmen einer dreijährigen Berufsausbildung 1 . Ab dem Jahr 2008 bestand zusätzlich die Option, freiwillig ein Studium der Hebammenwissenschaft ausbildungsintegriert oder nachqualifizierend zu absolvieren 1 . Basierend auf der EU-Richtlinie 2005/36/EG5 2 wurde die Berufsausbildung zur Hebamme im Jahr 2019 bundesweit in ein duales primärqualifizierendes Bachelorstudium transformiert.

Mit der Akademisierung wurde die theoretische Ausbildung von Berufsschulen an Hochschulen überführt, um den steigenden Anforderungen der geburtshilflichen Versorgung zu begegnen. Laut der International Confederation of Midwives (ICM) wird als Kompetenz die Mindestanforderung an Wissen, Fertigkeiten und beruflichen Verhalten definiert, die Hebammen für den Berufseinstieg benötigen 3 . Für das Studium in Deutschland sind verbindliche Kompetenzziele im Hebammengesetz (§ 9 HebG) sowie in der Studien- und Prüfungsverordnung (HebStPrV) definiert. Diese an das „Competency Framework“ der ICM angelehnten Kompetenzen werden im Rahmen des Studiums, in modularen Curricula mit steigender Komplexität, vermittelt.

Zusätzlich soll die Akademisierung dazu beitragen, die Attraktivität des Hebammenberufs zu steigern und so dem Fachkräftemangel entgegenzuwirken 4 5 . Die Anzahl der angebotenen Ausbildungs- und Studienplätze für angehende Hebammen wurde um ca. 54%, von 2.085 im Jahr 2011 auf 3.209 im Jahr 2023, gesteigert 6 7 . Mit der Steigerung wird den politischen Forderungen die Hebammenversorgung auszuweiten 8 , um den gesetzlichen Anspruch auf Hebammenhilfe (§ 134a SGB V) sowie die freie Wahl des Geburtsorts (§ 24f SGB V) für Frauen und Familien zu garantieren, gefolgt.

### Vorbereitung auf die Berufstätigkeit

Mit der Kombination aus theoretischen Kenntnissen und berufspraktischen Erfahrungen sollen Studierende durch das primärqualifizierende Studium der Hebammenwissenschaft für die Ausübung des Hebammenberufs in unterschiedlichen Tätigkeitsfeldern befähigt werden (§ 9 Abs. 1 HebG). Zudem sollten Studierende für die interprofessionelle Zusammenarbeit mit weiteren an der Versorgung beteiligten Berufsgruppen ausgebildet werden (§ 9 Abs. 4 HebG). Die theoretische Ausbildung erfolgt an Hochschulen (§ 3 HebStPrV), zum Zeitpunkt Februar 2024 an 46 Standorten 9 . Die berufspraktische Ausbildung erfolgt in klinischen sowie außerklinischen Einsätzen, die zum Zeitpunkt Juni 2024 durch 301 verantwortliche Praxiseinrichtungen 10 organisiert werden. Diese haben Kooperationen mit Krankenhäusern und hebammengeleiteten Einrichtungen wie Geburtshäusern, Praxen oder mit freiberuflichen Hebammen.

Für den individuellen Kompetenzerwerb werden Studierende zum einen durch Praxisbegleitungen (§ 17 HebG), punktuelle Begleitungen durch Mitarbeitende der Hochschulen zur Überprüfung des Theorie-Praxis Transfers während der berufspraktischen Einsätze, unterstützt. Zum anderen werden Studierende in 15–25% (§ 13 ff. HebG) der berufspraktischen Zeit durch Praxisanleitungen, Anleitungen in der Versorgung durch eine berufspädagogisch qualifizierte Hebamme, im individuellen Kompetenzerwerb unterstützt. Inwieweit sich Studierende der primärqualifizierenden Studiengänge durch das Studium auf die Ausübung des Hebammenberufs und die interprofessionelle Zusammenarbeit subjektiv vorbereitet fühlen und ob Unterschiede zwischen Studierenden höherer und niedriger Semester bestehen, wurde bisher in Deutschland nicht untersucht. Mit der Akademisierung sind Erkenntnisse zur Vorbereitung auf die Berufstätigkeit von Bedeutung, um bei Bedarf die theoretische und berufspraktische Ausbildung im Hebammenstudium iterativ anzupassen.

### Präferenzen für Tätigkeitsfelder

Mit dem Erwerb der Erlaubnis zum Führen der Berufsbezeichnung Hebamme können Studierende unmittelbar den Hebammenberuf angestellt und/ oder freiberuflich ausüben. Trotz der gesamtgesellschaftlichen Aufgabe von Hebammen, gibt es für die Hebammenversorgung in Deutschland keinen gesetzlich geregelten Sicherstellungsauftrag. Hebammen können somit ihre Tätigkeitsfelder frei nach persönlicher Präferenz und unabhängig vom Versorgungsbedarf von Frauen und Familien wählen. In Deutschland gibt es zudem kein zentrales Register, in dem Informationen zu beruflichen Biographien, Tätigkeitsfeldern, zeitlichen Umfängen und räumlichen Versorgungsgebieten von Hebammen bundesweit geführt werden. Es stehen ausschließlich Informationen zu den absoluten Zahlen festangestellter Hebammen in Krankenhäusern vom Statistischen Bundesamt (11.492 im Jahr 2023) 7 sowie spezifische Informationen zu den Mitgliedern des größten Berufsverbandes, dem Deutschen Hebammenverband (21.685 im Jahr 2022) 11 , zur Verfügung.

In welchen Tätigkeitsfeldern Studierende der Hebammenwissenschaft praktizieren möchten, wurde seit der Vollakademisierung in Deutschland bisher nicht untersucht. Das grundsätzliche Interesse an einer Tätigkeit in der direkten Versorgung zeigte eine australische Studie auf, in der 96% der Studierenden im letzten Studienjahr angaben, zukünftig als Hebamme praktizieren zu wollen 12 . Ähnliche Erkenntnisse zeigte eine Erhebung aus einem ausbildungsintegrierten Hebammenstudiengang in Deutschland, in der 91% der Absolvierenden angaben, nach dem Studium in der direkten Versorgung tätig zu sein. Davon waren 39% angestellt, 27% freiberuflich und 34% sowohl angestellt als auch freiberuflich tätig 13 . Eine quantitative Studie mit Auszubildenden und Studierenden ( *n* =644) aus Deutschland zeigte, dass insbesondere die außerklinischen Tätigkeitsfelder am häufigsten präferiert wurden 14 . Die klinischen Tätigkeitsfelder wurden, abgesehen von der Geburtshilfe (73%), nur von einem geringeren Anteil als interessant eingestuft 14 .

Studien deuten darauf hin, dass die Erfahrungen in den berufspraktischen Einsätzen Einfluss darauf nehmen, ob Studierende zukünftig als Hebamme praktizieren wollen 15 16 17 und welches Tätigkeitsfeld sie wählen 15 18 19 20 . Als Faktoren gegen die Wahl eines Tätigkeitsfelds wurden fehlende Wertschätzung und Mobbing 15 16 , Desillusionierung der Hebammentätigkeit 17 sowie ein eingeschränktes Verhältnis von Arbeit und Privatleben 17 19 von Studierenden identifiziert. Im Gegensatz dazu wurde eine Tätigkeit in einem kontinuierlichen Betreuungsmodell 19 als positiver Faktor für die Wahl eines Tätigkeitsfelds bewertet. In Anbetracht der Tatsache, dass 56% der Krankenhäuser Probleme bei der Besetzung von vakanten Hebammenstellen angeben 21 und im außerklinischen Setting Versorgungsengpässe zu verzeichnen sind 22 , gewinnen Erkenntnisse über die Präferenzen für Tätigkeitsfelder von Studierenden für die Organisation der Hebammenversorgung in Deutschland an Relevanz.

Ziel der Studie ist, erstmalig die Vorbereitung auf die Ausübung des Hebammenberufs und die interprofessionelle Zusammenarbeit durch das Studium sowie die Präferenzen für Tätigkeitsfelder von Studierenden zu untersuchen. Folgende Forschungsfragen wurden untersucht:

1. Wie fühlen sich Studierende in einem primärqualifizierenden Studiengang der Hebammenwissenschaft auf die Ausübung des Hebammenberufs vorbereitet und gibt es einen Unterschied zwischen Studierenden>5. Semester und≤5. Semester?
2. Wie fühlen sich Studierende in einem primärqualifizierenden Studiengang der Hebammenwissenschaft auf die interprofessionelle Zusammenarbeit vorbereitet und gibt es einen Unterschied zwischen Studierenden>5. Semester und≤5. Semester?
3. In welchen Tätigkeitsfeldern möchten Studierende der Hebammenwissenschaft präferiert arbeiten?

## Methodik

### Studiendesign

Die „Healthy MidStudents“-Studie ist eine explorative Querschnittsstudie, in der die Gesundheits- und Arbeitssituation von Studierenden der Hebammenwissenschaft in Norddeutschland untersucht wurde. In diesem Artikel werden Erkenntnisse zur Vorbereitung auf die Ausübung des Hebammenberufs und interprofessionelle Zusammenarbeit sowie die Präferenzen für Tätigkeitsfelder präsentiert. Registriert wurde die Studie beim Open Science Framework (OSF) 23 .

### Studienpopulation

Die Studie wurde an neun Hochschulstandorten in fünf Bundesländern in Norddeutschland (Bremen, Hamburg, Mecklenburg-Vorpommern, Niedersachsen, Schleswig-Holstein) durchgeführt. Als Einschlusskriterium galt die Einschreibung in ein primärqualifizierendes, ausbildungsintegriertes oder nachqualifizierendes Studium der Hebammenwissenschaft. Studierende aus einem primärqualifizierenden Studiengang wurden ab dem 2. Semester eingeschlossen, um berufspraktische Erfahrungen voraussetzen zu können.

### Fragebogen

Der online-basierte Fragebogen bestand aus validierten Instrumenten und selbsterstellten Fragen. Einzelne Fragen konnten ausgelassen werden, da kein Item im Fragebogen verpflichtend war. Vorbereitend wurde ein Pre-Test mit drei wissenschaftlichen Mitarbeitenden durchgeführt. Das Geschlecht wurde mit einem Item von der deutschen Version vom Copenhagen Psychosocial Questionnaire III 24 und der Familienstand mit einem Item vom Mikrozensus 2023 erhoben 25 . Zur Erhebung von Alter, Migrationshintergrund, höchstem Bildungsstand und Elternstatus wurden selbsterstellte Fragen verwendet.

### Vorbereitung auf die Berufstätigkeit

Angesichts fehlender bestehender Instrumente zur Vorbereitung auf die Ausübung des Hebammenberufs sowie die interprofessionelle Zusammenarbeit wurden zwei selbsterstellte Fragen zur Selbsteinschätzung genutzt: „Wie fühlen Sie sich durch Ihr Studium auf den Hebammenberuf vorbereitet?“ und „Wie fühlen Sie sich durch Ihr Studium auf die interprofessionelle Zusammenarbeit vorbereitet?“. Folgende Antwortoptionen auf einer Likert-Skala waren verfügbar: (1=sehr schlecht bis 5=sehr gut).

### Präferenzen für Tätigkeitsfelder

**Abb. 1:**
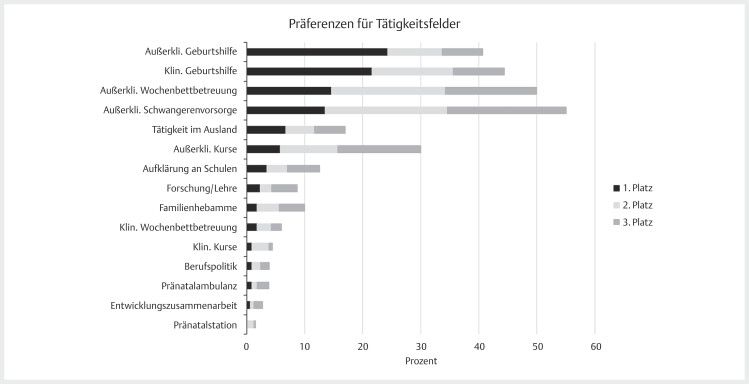
Darstellung der Präferenzen für Tätigkeitsfelder (1–3. Platz) ( *n* =342). Anmerkungen. Die Tätigkeitsfelder konnten für die Plätze 1–15 gewichtet werden. Die Ergebnisse sind nach der Gewichtung vom 1. Platz geordnet. Außerkli=Außerklinisch, Klin=Klinisch. Absolute und relative Häufigkeiten sind der Tab. 3 zu entnehmen.

Mithilfe einer Literaturrecherche wurden 15 Tätigkeitsfelder von Hebammen identifiziert ( Abb. 1 ). Die Teilnehmenden wurden gebeten, diese dahingehend zu gewichten, in welchen Tätigkeitsfeldern die Studierenden zum jetzigen Zeitpunkt präferiert praktizieren wollen würden (1. Platz=größtes Interesse bis 15. Platz=geringstes Interesse). Zusätzlich wurde der Ausfüllhinweis, dass nicht alle Items verwendet werden müssen, gegeben.

### Datenerhebung

Die Datenerhebung wurde vom 17.10.2022 bis 31.01.2023 durchgeführt. Rekrutiert wurden Teilnehmende in Einverständnis mit den Studiengangsleitungen während Lehrveranstaltungen in Präsenz, online oder via E-Mail. Ergänzend wurde eine Erinnerung von den Studiengangsleitungen an die Studierenden versendet. Die Rücklaufquote beläuft sich mit insgesamt 343 Teilnehmenden bei einer Gesamtpopulation von 560 Studierenden auf 61,3%. Eine Teilnehmende wurde ausgeschlossen, da das Ausschlusskriterium<2. Semester vorlag und daher keine berufspraktischen Erfahrungen garantiert waren ( *n* =342). Die Teilnahme war freiwillig, anonym und es wurden keine Incentives vergeben.

### Datenauswertung

Die Auswertung der Studienergebnisse erfolgte deskriptiv unter Angabe von absoluten ( *n* ) und relativen ( *%* ) Häufigkeiten sowie der Berechnung von Mittelwerten ( *M* ) und Standardabweichungen ( *SD* ). Gruppenvergleiche wurden mit einem zweiseitigen *t* -Test für unabhängige Stichproben durchgeführt. Das Signifikanzniveau wurde auf *p* <0,05 und das Konfidenzintervall auf 95% festgelegt. In der Analyse zur Vorbereitung auf die Berufstätigkeit wurden Studierende aus ausbildungsintegrierten und nachqualifizierenden Studiengängen sowie im Urlaubssemester ausgeschlossen. Die statistische Analyse erfolgte mit IBM SPSS Statistics v. 26 (IBM, Armonk, NY, USA).

### Ethik

Die Studie wurde nach dem Erhalt von einem positiven Ethikvotum der Lokalen Ethikkommission am Universitätsklinikum Hamburg-Eppendorf (LPEK-0505) und im Einklang mit der Deklaration von Helsinki durchgeführt. Die Teilnehmenden haben vor Studienteilnahme schriftlich ihre Einverständniserklärung gegeben.

## Ergebnisse

### Deskriptive Charakteristika

Die soziodemografischen Charakteristika der Stichprobe ( *n* =342) können der Tab. 1 entnommen werden. Die Teilnehmenden waren zum Großteil weiblich (99,1%), zwischen 21–30 Jahre alt (74,8%), ohne Migrationshintergrund (88,3%), ledig (84,2%) und kinderlos (85,1%). Die Mehrheit verfügte über die Hochschulreife als höchsten Bildungsstand (57,3%) und war im 3. Semester (44,4%) immatrikuliert.

**Table:** **Tab. 1** Soziodemografische Charakteristika der Stichprobe ( *n* =342).

Variable	*n*	%
Geschlecht ( *n* =342)		
Weiblich	339	99,1
Männlich	1	0,3
Divers	2	0,6
Alter in Jahren ( *n* =338)		
≤20	35	10,3
21–30	256	74,8
31–40	37	10,9
≥41	10	3,0
Migrationshintergrund ( *n* =342)		
Ja	40	11,7
Nein	302	88,3
Familienstand ( *n* =341)		
Ledig	288	84,2
Verheiratet	45	13,2
Geschieden	7	2,0
Eingetragene Partnerschaft	1	0,3
Höchster Bildungsgrad ( *n* =342)		
Hochschulreife	195	57,3
Berufsausbildung	84	24,5
Bachelor	43	12,6
Master	17	4,7
Diplom	3	0,9
Promotion	0	0,0
Semester ( *n* =341)		
2.	1	0,3
3.	152	44,4
4.	27	7,9
5.	120	35,1
6.	10	2,9
7.	29	8,5
8.	1	0,3
Urlaubssemester	1	0,3
Art des Studiengangs *(n* =341)		
primärqualifizierend	253	74,0
ausbildungsintegriert	81	23,7
nachqualifizierend	7	2,0

### Vorbereitung auf die Berufstätigkeit

Studierende in einem primärqualifizierenden Studiengang bewerteten ihre Vorbereitung auf die Ausübung des Hebammenberufs ( *n* =249) im Durchschnitt als mittelmäßig ( Tab. 2 ). Insgesamt fühlten sich 5,6% sehr gut, 31,3% gut, 47,0% mittelmäßig, 13,7% schlecht und 2,4% sehr schlecht auf die Ausübung des Hebammenberufs durch das Studium vorbereitet. Beim Gruppenvergleich bewerteten Studierende<5. Semester die Vorbereitung auf die Ausübung des Hebammenberufs deskriptiv geringfügig besser als Studierende≥5. Semester, ohne statistisch signifikanten Unterschied ( *d* =0,15; *p* =0,25).

**Table:** **Tab. 2** Gruppenvergleich der Vorbereitung auf die Ausübung des Hebammenberufs und die interprofessionelle Zusammenarbeit von primärqualifizierenden Studierenden ( *n* =249).

Variable	*n*	*M*	*SD*	*ΔM (95% -CI)*	*d*	*p*
Vorbereitung auf die Ausübung des Hebammenberufs								
<5. Semester	143	3,29	0,82	0,12 (-0,09; 0,34)	0,15	0,25
≥5. Semester	106	3,22	0,76			
Vorbereitung auf die interprofessionelle Zusammenarbeit								
<5. Semester	143	3,22	0,76	-0,17 (-0,37; 0,02)	-0,22	0,08
≥5. Semester	106	3,40	0,77			

**Anmerkungen.** M=Mittelwert, SD=Standardabweichung, ΔM=Mittelwertdifferenz, CI=Konfidenzintervall, d=Cohens d, p=p-Wert, Antwortoptionen: 1=sehr schlecht, 2=schlecht, 3=mittelmäßig, 4=gut, 5=sehr gut.

Auch die Vorbereitung auf die interprofessionelle Zusammenarbeit ( *n* =249) wurde im Durchschnitt als mittelmäßig bewertet ( Tab. 2 ). Die Studierenden gaben an, dass sich 3,2% sehr gut, 37,3% gut, 47,4% mittelmäßig, 10,0% schlecht und 2,0% sehr schlecht auf die interprofessionelle Zusammenarbeit vorbereitet fühlten. Die Vorbereitung auf die interprofessionelle Zusammenarbeit wurde von Studierenden des≥5. Semester deskriptiv geringfügig besser als von Studierenden<5. Semester bewertet, ohne statistisch signifikanten Unterschied ( *d* =−0,22; *p* =0,08).

### Präferenzen für Tätigkeitsfelder

In der Abb. 1 und Tab. 3 werden die Präferenzen der Studierenden für die Tätigkeitsfelder dargestellt. Die Ergebnisse zeigen, dass die außerklinische Geburtshilfe (24,3%), gefolgt von der klinischen Geburtshilfe (21,6%) und der außerklinischen Wochenbettbetreuung (14,6%) am häufigsten von Studierenden auf dem 1. Platz gewichtet wurden ( *n* =342).

**Table:** **Tab. 3** Übersicht der Präferenzen für Tätigkeitsfelder mit absoluten und relativen Häufigkeiten ( *n* =342).

Tätigkeitsfeld	1. Platz	2. Platz	3. Platz	4.- 15. Platz	Nicht gewichtet
	*n* (%)				
Außerklinische Geburtshilfe	83 (24,3)	32 (9,4)	24 (7,0)	69 (20,2)	134 (39,2)
Klinische Geburtshilfe	74 (21,6)	48 (14,0)	30 (8,8)	82 (24,0)	108 (31,6)
Außerklinische Wochenbettbetreuung	50 (14,6)	67 (19,6)	54 (15,8)	95 (27,8)	76 (22,2)
Außerklinische Schwangerenvorsorge	46 (13,5)	72 (21,1)	70 (20,5)	73 (21,3)	81 (23,7)
Tätigkeit im Ausland	23 (6,7)	17 (5,0)	18 (5,3)	84 (24,6)	200 (58,5)
Außerklinische Kurse	20 (5,8)	34 (9,9)	49 (14,3)	128 (37,4)	111 (32,5)
Aufklärung an Schulen	12 (3,5)	12 (3,5)	19 (5,6)	123 (36,0)	176 (51,5)
Forschung und/oder Lehre	8 (2,3)	7 (2,0)	15 (4,4)	82 (24,0)	230 (67,3)
Klinische Wochenbettbetreuung	6 (1,8)	8 (2,3)	7 (2,0)	54 (15,8)	267 (78,1)
Familienhebamme	6 (1,8)	13 (3,8)	15 (4,4)	85 (24,9)	223 (65,2)
Pränatalambulanz	3 (0,9)	3 (0,9)	7 (2,0)	63 (18,4)	266 (77,8)
Klinische Kurse	3 (0,9)	10 (2,9)	2 (0,6)	74 (21,6)	253 (74,0)
Berufspolitik	3 (0,9)	5 (1,5)	5 (1,5)	67 (19,6)	262 (76,6)
Entwicklungszusammenarbeit	2 (0,6)	2 (0,6)	5 (1,5)	56 (16,4)	277 (81,0)
Pränatalstation	0 (0)	4 (1,2)	1 (0,3)	42 (13,7)	295 (86,3)

**Anmerkungen** . Die Gesamtsummen können aufgrund von Rundungsdifferenzen nicht 100% ergeben.

Für die präferierten Tätigkeitsfelder zeigt die kumulierte Betrachtung der ersten drei Plätze, dass die außerklinische Schwangerenvorsorge (55,0%), gefolgt von der außerklinischen Wochenbettbetreuung (50,0%) und klinischen Geburtshilfe (44,1%) von Studierenden am häufigsten gewichtet wurde. Die weiteren klinischen Tätigkeitsfelder, Wochenbettbetreuung (6,1%), Kurse (4,4%), Pränatalambulanz (3,8%) und -station (1,5%) wurden von Studierenden kumuliert betrachtet selten auf den ersten drei Plätzen gewichtet.

Zehn von fünfzehn der Tätigkeitsfelder wurden vom Großteil der Studierenden (>50%) nicht in der Gewichtung der präferierten Tätigkeitsfelder aufgeführt ( Tab. 3 ). Insbesondere die Tätigkeitsfelder Pränatalstation (86,3%), Entwicklungszusammenarbeit (81,0%) und klinische Wochenbettbetreuung (78,1%) wurden vom Großteil der Studierenden gar nicht als präferierte Tätigkeitsfelder gewichtet.

## Diskussion

Mit dieser Studie konnten die ersten Erkenntnisse zur Vorbereitung auf die Ausübung des Hebammenberufs und die interprofessionelle Zusammenarbeit von Studierenden der primärqualifizierenden Studiengänge sowie die Präferenzen für Tätigkeitsfelder von Studierenden, unabhängig der Studienform, gewonnen werden.

### Vorbereitung auf die Berufstätigkeit

Studierende der Hebammenwissenschaft fühlten sich im Durchschnitt mittelmäßig auf die Ausübung des Hebammenberufs vorbereitet. Angesichts des globalen Items kann die Vorbereitung auf die Ausübung des Hebammenberufs nur übergeordnet und nicht spezifisch für die einzelnen Hebammenkompetenzen interpretiert werden. Die Tatsache, dass die Studierenden>5 Semester ihre Vorbereitung auf die Ausübung des Hebammenberufs durch das Studium ähnlich wie Studierende≤5 Semester bewerteten, ist überraschend. Ursächlich können die größere berufspraktische Erfahrung sowie die steigenden Anforderungen an die Studierenden in den Praxiseinsätzen sein. Auch Erfahrungen in der Zusammenarbeit mit Hebammen sowie die Unterstützung innerhalb der Organisation während der berufspraktischen Ausbildung können den Kompetenzerwerb von Studierenden fördern oder hemmen 26 27 28 . Ergänzend können der zunehmende gesundheitsbezogene Wissensstand sowie Erfahrungen mit seltenen geburtshilflichen Komplikationen dazu führen, dass sich Studierenden selbstkritischer bewerten. Eine Studie mit Studierenden aus Schweden zeigte, dass sich Studierende für die Versorgung in der Schwangerschaft, während der Geburt, im Wochenbett sowie des Neugeborenen im Durchschnitt kompetent fühlten 29 . Demgegenüber gaben die Studierenden größere Unsicherheiten in der Versorgung von Frauen mit regelwidrigen Verläufen im Vergleich zu physiologischen Verläufen an 29 .

Auch die globale Vorbereitung auf die interprofessionelle Zusammenarbeit wurde von den Studierenden durchschnittlich als mittelmäßig bewertet. Ursächlich können die bisher primär monoprofessionell organisierten Studiengänge der Hebammenwissenschaft mit nur geringfügig etablierten interprofessionellen Lehr-Lern Angebote mit Studierenden der Medizin, Pflege und weiteren Professionen sein 30 . Auch das Ergebnis, dass kein statistischer Unterschied in der Einschätzung der Vorbereitung auf die interprofessionelle Zusammenarbeit im Ausbildungsstand lag, könnte durch das geringe Angebot an Veranstaltungen sowie Erfahrungen aus der berufspraktischen Ausbildung bedingt sein. Die Relevanz der interprofessionellen Zusammenarbeit wird durch die politische Forderung im Nationalen Gesundheitsziel, dass „ *die an der Versorgung beteiligten Berufsgruppen [..] konstruktiv und partnerschaftlich* “ 31 zusammenarbeiten, hervorgehoben. Diese Zusammenarbeit ist nicht nur entscheidend für die Patient:innensicherheit von Mutter und Kind, sondern auch für die erfolgreiche Implementierung von Versorgungsmodellen wie dem hebammengeleiteten Kreißsaal. Die Ausweitung von interprofessionellen Lehr-Lern Veranstaltungen sowie Maßnahmen zur Förderung der interprofessionellen Zusammenarbeit in der Versorgung, wären zu empfehlen.

Zur differenzierteren Betrachtung der Vorbereitung auf die Ausübung des Hebammenberufs und die interprofessionelle Zusammenarbeit könnten zukünftig neben einem globalen Item detaillierte Instrumente zum Erheben der spezifischen Hebammenkompetenzen von Pehlke-Milde 32 oder adaptiert von Kranz et al. 33 verwendet werden. Zusätzlich wären zur Einordnung der Ergebnisse Studien empfehlenswert, die die subjektive Einschätzung mit objektiven Performanzmessungen, z. B. OSCE (Objective structured clinical examination) 34 , vergleichen. Zudem sollte qualitativ untersucht werden, welche Faktoren die Vorbereitung beeinflussen und welche Maßnahmen diese stärken würden. Übergeordnet bieten sich Portfolios für die engmaschige Evaluation des individuellen Lernfortschritts für Studierende an. Zusätzlich könnte die Implementierung von Mentoring Programmen die individuelle Berufsvorbereitung fördern 28 29 .

### Präferenzen für Tätigkeitsfelder

Die Gewichtung der Tätigkeitsfelder zeigt, dass die Mehrheit der Studierenden Interesse an der direkten Versorgung angab. Das große Interesse an der praktischen Hebammenversorgung geht mit Erkenntnissen zu Studierenden aus Australien 12 und Absolvierenden aus Deutschland einher 13 . Parallel zeigte sich eine Präferenz für nur wenige ausgewählte Tätigkeitsfelder, da der Großteil der Studierenden (>50%) zehn der insgesamt fünfzehn Tätigkeitsfelder gar nicht bei der Gewichtung berücksichtigte. Übergeordnet zeigte sich eine große Präferenz für die originären Tätigkeitsfelder von Hebammen (Schwangerenvorsorge, Geburtshilfe, Wochenbettbetreuung) durch die Studierenden. Die Tatsache, dass 45,9% der Studierenden die Geburtshilfe unabhängig vom Betreuungssetting auf dem ersten Platz gewichteten, zeigt das Interesse an der geburtshilflichen Hebammenarbeit.

Übergeordnet zeigte sich in der vorliegenden Studie eine größere Präferenz für die außerklinischen als für die klinischen Tätigkeitsfelder. Die Gewichtung der außerklinischen Geburtshilfe auf dem ersten Platz (24,3%) durch die Studierenden ist angesichts der hohen, nicht erfüllten Nachfrage an Haus- und Geburtshausgeburten 22 und des Rechts auf freie Wahl des Geburtsorts (§ 24f SGB V) positiv zu bewerten. Auch die Präferenz von 14,6% der Studierenden, die die außerklinische Wochenbettbetreuung auf dem ersten Platz gewichteten, ist für die Versorgungsengpässe in der Wochenbettbetreuung aussichtsreich 22 .

Die kumulierte Betrachtung der ersten drei Plätze zeigt, dass die außerklinische Schwangerenvorsorge von 55,1% der Studierenden als Präferenz gewichtet wurde. Das Ergebnis kommt der politischen Strategie zum Nationalen Gesundheitsziel „Gesundheit rund um die Geburt“ 31 , in dem zur Förderung einer gesunden Schwangerschaft, die multiprofessionelle Schwangerenvorsorge gefordert wird, entgegen. Diese Ergebnisse der vorliegenden Studie gehen mit Erkenntnissen einer Erhebung aus Deutschland einher, in der auch die außerklinischen Tätigkeitsfelder, insbesondere die Schwangerenvorsorge und die Wochenbettbetreuung, von Hebammenauszubildenden und –studierenden mehrheitlich präferiert wurden 14 . Die Präferenz in einem kontinuierlichen Betreuungsmodell zu praktizieren, welches in der außerklinischen Tätigkeit häufiger gegeben ist 35 , könnte die Gewichtung der Studierenden in der vorliegenden Studie begründen. Studien mit Studierenden und examinierten Hebammen zeigten bereits den Wunsch nach einer Tätigkeit in einem kontinuierlichen Betreuungsmodell auf 12 36 , was im Einklang mit den Hinweisen der vorliegenden Studie ist.

Die Tätigkeit in der klinischen Geburtshilfe wurde, ähnlich wie die außerklinische Geburtshilfe, mit hoher Präferenz auf dem ersten Platz (21,6%) und in der kumulierten Betrachtung auf den ersten drei Plätzen (44,4%) gewichtet. Unter Berücksichtigung der Rate an unbesetzten Hebammenstellen in Krankenhäusern 21 und des nicht erfüllten Bedarfs an Beleggeburten 22 , ist das Interesse positiv zu beurteilen. Da in der vorliegenden Studie nicht erfragt wurde, ob Studierende die klinische Geburtshilfe angestellt oder freiberuflich ausüben wollen würden, bleibt offen, ob ein hebammen- oder ärztlichgeleitetes Betreuungsmodell präferiert werden würde. Als Tätigkeitsfeld in einem hebammengeleiteten Betreuungsmodell kommen neben der Tätigkeit als Beleghebamme auch eine Tätigkeit in einem hebammengeleiteten Kreißsaal in Frage, deren Ausweitung angesichts des positiven Nutzens für die Versorgungsqualität bereits gefordert wird 5 . Zudem zeigen nationale Studien, dass die Bereitschaft von examinierten Hebammen zur Rückkehr in die klinische Geburtshilfe durch die Implementierung von hebammengeleiteten Kreißsälen steigt 37 38 .

Im Kontrast dazu steht die geringe Gewichtung der weiteren klinischen Tätigkeitsfelder, abgesehen von der Geburtshilfe, durch die Studierenden. Ein geringer Anteil der Studierenden gewichtete die Tätigkeitsfelder Pränatalambulanz (3,8%) und -station (1,5%), Kurse (4,4%) sowie klinische Wochenbettbetreuung (6,1%) kumuliert betrachtet auf den ersten drei Plätzen, obwohl diese Tätigkeitsfelder analoge Tätigkeiten zur außerklinischen Versorgung beinhalten. Das Ergebnis geht mit Erkenntnissen aus einer nationalen Erhebung einher, in der die Pränatalstation, -ambulanz und Kurse von den angehenden Hebammen als am uninteressantesten eingestuft wurden 14 . Insbesondere die geringe Gewichtung der klinischen Wochenbettbetreuung rückt in den Fokus, wenn man berücksichtigt, dass die „Überwachung des Wochenbettverlaufs“ eine vorbehaltene Tätigkeit von Hebammen ist (§4 HebG). Die Rahmenbedingungen der Schwangerenvorsorge und Wochenbettbetreuung durch Hebammen variieren zwischen dem außerklinischen und klinischen Setting. Zum einen erfolgt die Versorgung im klinischen Setting nicht mono- sondern multiprofessionell, sodass eine unzureichende Vorbereitung auf die interprofessionelle Zusammenarbeit sowie eine negativ erlebte interprofessionelle Zusammenarbeit 39 die Präferenz für das Setting beeinflussen könnte. Zum anderen sind abgesehen von hebammengeleiteten Kreißsälen, die Pränatalambulanz und –station sowie die Wochenstation in deutschen Krankenhäusern ärztlich geleitet, sodass die berufliche Autonomie 19 40 von Hebammen eingeschränkt sein kann. Das Fehlen von kontinuierlichen hebammengeleiteten Betreuungsmodellen im Krankenhaus kann eine weitere Ursache für die untergeordnete Bedeutung der weiteren klinischen Tätigkeitsfelder darstellen. Die Erkenntnisse einer Studie mit examinierten Hebammen in Deutschland untermauern die Präferenz von Hebammen, einer Tätigkeit in einem Hebammen- statt einem ärztlich geleiteten Betreuungsmodell nachzugehen 37 . Darüber hinaus liegen Studien vor, die zeigen, dass Hebammen, die im Krankenhaus praktizieren, selten auf Wochen- und Pränatalstationen sowie in der Pränatalambulanz tätig sind 41 42 . Dies könnte dazu führen, dass es Studierenden an Hebammen-Vorbildern mangelt, was wiederum ihre Präferenz beeinflussen könnte. Des Weiteren unterscheidet sich die Zielgruppe der Hebammenversorgung in den klinischen Tätigkeitsfeldern dahingehend, dass im klinischen Setting erheblich mehr Schwangere, Gebärende und Wöchner:innen mit geburtshilflichen Risiken betreut werden.

Nur ein geringer Anteil der Studierenden (8,7%) hat das Tätigkeitsfeld Forschung und/ oder Lehre kumuliert betrachtet auf den ersten drei Plätzen gewichtet. Die Ergebnisse der vorliegenden Studie widerlegen somit die Befürchtung, dass der Großteil der akademisierten Hebammen eine Tätigkeit in der Wissenschaft gegenüber der praktischen Versorgung bevorzugen würde. Gleichzeitig besteht für die Disziplinentwicklung und Fortführung hebammenwissenschaftlicher Forschung ein großer Bedarf an qualifiziertem akademischem Nachwuchs 43 44 . Dabei sind berufspraktische Erfahrungen für eine zukünftige wissenschaftliche Tätigkeit von Vorteil. Um diesen Bedarf zu decken, ist es empfehlenswert, sowohl die Sichtbarkeit von hebammenwissenschaftlicher Forschung zu erhöhen als auch die Optionen zur Weiterqualifikation (Master/ Promotion) zu stärken 43 .

Insbesondere um die vergleichsweise geringe Gewichtung für klinische Tätigkeitsfelder, abgesehen von der Geburtshilfe, näher einordnen zu können, sind qualitative Studien zur Untersuchung der Faktoren zur Wahl der zukünftigen Tätigkeitsfelder sinnvoll. Ferner bieten Kohortenstudien die Chance, einen Überblick über die beruflichen Biographien von akademisierten Hebammen zu gewinnen und zu eruieren, inwieweit sich die Präferenzen über das Studium und den Berufseinstieg hinweg verändern. Parallel wurden bereits erste berufspolitische Forderungen an ein nationales Hebammenregister, zum Monitoring der praktizierenden Hebammen in Deutschland, formuliert 5 45 46 . Zudem sollte für die Versorgungsplanung evaluiert werden, ob die Tätigkeiten von Hebammen mit dem bundesweiten Versorgungsbedarf von Frauen und Familien kongruent ist.

### Limitationen

Basierend auf dem Querschnittsdesign kann es zu einem Selektionsbias gekommen sein. Zudem kann es angesichts der COVID-19-Pandemie zu Einschränkungen in der Lehre und Versorgung gekommen sein, dass die Vorbereitung auf die Ausübung des Hebammenberufs sowie auf die interprofessionelle Zusammenarbeit eingeschränkt war. Dass einige Tätigkeitsfelder von der Mehrheit der Studierenden nicht gewichtet wurden, erschwert die Interpretation der Ergebnisse. Als mögliche Ursachen hierfür können mangelndes Interesse oder eine unklare Meinungsbildung der Studierenden angeführt werden. Ein möglicher Erklärungsansatz für die unklare Meinungsbildung könnte in fehlenden Berührungspunkten der Studierenden mit den betreffenden Tätigkeitsfeldern, wie beispielsweise der Entwicklungszusammenarbeit, liegen. Darüber hinaus wurden Tätigkeitsfelder wie Kreißsaalleitung oder Praxisanleitung nicht erfragt, sodass es hier zu Verzerrungen gekommen sein kann. Basierend auf dem Rücklauf von 61,3% kann allerdings von einer hohen Repräsentativität der Ergebnisse für die Gesamtpopulation ausgegangen werden.

## Schlussfolgerungen

Die Studie liefert erste wichtige Erkenntnisse zur Berufsvorbereitung und die Präferenzen für Tätigkeitsfelder von Studierenden. Die subjektive Bewertung einer mittelmäßigen Vorbereitung auf die Ausübung des Hebammenberufs und die interprofessionelle Zusammenarbeit von Studierenden der primärqualifizierenden Studiengänge sollte mit unterschiedlichen wissenschaftlichen Methoden näher beleuchtet werden. Die Studienergebnisse zeigen, dass der Großteil der Studierenden eine Tätigkeit in der außerklinischen Schwangerenvorsorge, der außerklinischen und klinischen Geburtshilfe sowie in der außerklinischen Wochenbettbetreuung präferiert.
